# Supplementary material for: Next generation biosecurity: Towards genome based identification to prevent spread of agronomic pests and pathogens using nanopore sequencing
Source: PLoS One. 2022 Jul 25;17(7):e0270897. doi: 10.1371/journal.pone.0270897 (PMC9312391; doi:10.1371/journal.pone.0270897)
Supplement: S1 File — dx.doi.org/10.17504/protocols.io.bx7nprme. (PDF) [file pone.0270897.s001.pdf]

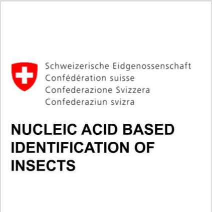

WORKFLOW FOR THE NUCLEIC ACID BASED IDENTIFICATION OF INSECTS USING WHOLE GENOME AMPLIFICATION AND NANOPORE SEQUENCING - Monarch®

Jürg E Frey[1], Beatrice Frey[2], Daniel Frei[2,3], Morgan Gueuning[2,4], Simon Blaser[5,6], Andreas Bühlmann[7]

[1]Agroscope;
[2]Agroscope, Department of Method Development and Analytics, Research Group Molecular Diagnostics, Genomics and Bioinformatics, Wädenswil, Switzerland;
[3]Current address: Qiagen Instruments AG, Hombrechtikon, Switzerland;
[4]Current address: Department of Research and Development, Blood Transfusion Service Zurich, Swiss Red Cross, Schlieren, Switzerland;
[5]Agroscope, Department of Plants and Plant Products, Agroscope Phytosanitary Service;
[6]Current address: Forest Health and Biotic Interactions, Federal Research Station for Forest, Snow and Landscape, Birmensdorf, Switzerland;
[7]Agroscope, Department of Plants and Plant Products, Research Group Product Quality and Innovation

dx.doi.org/10.17504/protocols.io.bx7nprme

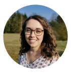

Julia Rossmanith
protocols.io

This protocol uses the Monarch® Genomic DNA Purification Kit for DNA extraction and purification.
An alternative version that uses a generic Proteinase K buffer (KAWA buffer) for extraction can be found here:

WORKFLOW FOR THE NUCLEIC ACID BASED IDENTIFICATION OF INSECTS USING WHOLE GENOME AMPLIFICATION AND NANOPORE SEQUENCING - KAWA
by Julia Rossmanith

BACKGROUND

World-wide trade with plant material has dramatically increased over the past decades, and with it has the risk for accidental introduction of potential plant pests and diseases. Rapid and accurate nucleic acid based identification of such quarantine organisms has become an important tool to minimize their dispersal.
Nucleic acid based identification exploits genetic diversity. A basic tenet holds that species generally do not interbreed and hence, the level of genetic differentiation within species is generally lower than between species. For insects, this pattern of genetic diversity is being used by DNA barcoding with great success. An approximately 600 base pairs long fragment in the first half of the mitochondrially encoded cytochrome c oxidase I gene (COI) is used as a reference sequence to identify insects at the species level. So far, reference sequences for ca. 231’000 different insect species are deposited on the publicly available “Barcoding for Life Database” BOLD (http://www.barcodinglife.org/; by June 2021). Another important database especially targeting phytosanitary purposes with DNA barcodes of vouchered reference specimen is the EPPO Q-Bank (European Plant Protection Organization, https://qbank.eppo.int/).

The methodology of DNA barcoding generally relies on PCR amplification of the diagnostic COI gene fragment using a pair of primers for which the exact DNA sequence must be known. However, this information is not always available, for example in the case of so far undescribed species or in cases, where genetic variation within species affected primer sites. Furthermore, although the COI marker sequence shows an impressive degree of among species differentiation, this is not true for all species and hence, a number of important pest species cannot be differentiated based on this marker alone.

An ideal method for species identification should therefore obtain information of the best discriminating genetic region or of several genetic regions. Here, we describe a state-of-the-art method to achieve this task.

PURPOSE

The purpose of this workflow is to provide a generic method for genetic identification of potential insect quarantine species and of other especially dangerous pest species in support of the Swiss Federal Plant Protection Service. The method is marker independent and may be used with reference databases of any genetic fragment. It is based on whole genome amplification, followed by single strand nanopore sequencing and DNA barcoding based identification.

LB_MM2_PcRefSeq_NrSeq_ret30_210212.geneiousWorkflow

RefDB_BOLD59kGBCocc755_60625Seq_211213.fasta

DOI

dx.doi.org/10.17504/protocols.io.bx7nprme

Jürg E Frey, Beatrice Frey, Daniel Frei, Morgan Gueuning, Simon Blaser, Andreas Bühlmann . WORKFLOW FOR THE NUCLEIC ACID BASED IDENTIFICATION OF INSECTS USING WHOLE GENOME AMPLIFICATION AND NANOPORE SEQUENCING - Monarch®. **protocols.io** https://dx.doi.org/10.17504/protocols.io.bx7nprme

insects, identification of insects, whole genome amplification, nanopore sequencing, nucleic acid

CC BY protocol ,

Sep 13, 2021

Jan 10, 2022

53198

SCOPE

This method is suitable for the qualitative identification of DNA (deoxyribonucleic acid) or cDNA (reverse-transcribed DNA of RNA of, e.g., viruses) of potentially all organisms. It has been tested against a broad taxonomic range of pest species. The workflow is designed to work with fresh, ethanol (EtOH; preferably 70%) preserved and frozen samples. The workflow presented here is established for insect species identification, but it was successfully applied to the identification of fungi and bacteria using the proper reference databases.

DEFINITIONS & ABBREVIATION

DNA: Deoxyribonucleic acid
PCR: Polymerase Chain Reaction
SOP: Standard Operating Procedure
UV: Ultraviolet
WGA: Whole Genome Amplification
CO1/COI: Mitochondrial *cytochrome c oxidase 1* gene
Bp: Base pairs

PRINCIPLE OF THE METHOD

The workflow starts with nucleic acid extraction (DNA and/or RNA followed by cDNA production), followed by a WGA step of the extracted DNA/cDNA, then a

clean-up step before producing the nanopore sequencing library which finally is loaded on the MinION nanopore sequencing device.

Several commercial kits were successfully used for nucleic acid extraction (mentioned in Section 'Materials') yet the best results for our workflow, both in terms of the number of active nanopores as well as the time to sequencing 200k reads, was obtained if a clean-up step was added after the extraction step. Here, we describe a workflow based on NEB's Monarch line for DNA extraction (New England Biolabs, Monarch Genomic DNA Purification Kit (Bioconcept AG, Allschwil, Switzerland, Order Nr. T3010S) using the provider's 'Protocol for Extraction and Purification of Genomic DNA from Tissues', followed by a reaction cleanup step using the Qiagen DNeasy PowerClean CleanUp Kit (Qiagen Instruments AG, Hombrechtikon, Switzerland, Order Nr. 12877-50).

The methods chosen for this workflow aim at flexibility of input material (gDNA, cDNA) and optimal output to enable multiple use of single flowcells. The workflow should in principle enable at least 10 individual runs of ca. 2-4 hours data collection on a single MinION flowcell.

The resulting sequence data, ideally > 200’000 reads per individual sample, are loaded into the software ‘Geneious Prime’ and analyzed by a custom-made workflow using the proper reference library (see section "Materials").

7 Raw Data Processing and Analysis

7.1 Primary Data Acquisition and Basecalling

Primary data acquisition is performed with the Oxford Nanopore Technologies (ONT) data acquisition software MinKNOW GUI v.4.2.8, the operating software that drives nanopore sequencing devices. Basecalling of the raw nanopore sequencing read data is required to generate the fastq sequence data files needed for further analysis. Basecalling is a time-consuming process and hence it is beneficial to use graphic processing units (GPU) to support this process. To enable use of GPU for basecalling generally requires access to a Linux based operating system with decent memory and storage capacity. We use a Dell Precision 7920 Tower XCTO Base with 256GB RAM and a Nvidia Quadro RTX6000, 24 GB graphic card running Ubuntu 18.04. We use the software Guppy v. 4.5.4 for basecalling using GPU with a parameter set established by Miles Benton (https://gist.github.com/sirselim/2ebe2807112fae93809aa18f096dbb94#file-basecalling_notes-md) to be used from a terminal window:
/guppy_basecaller --disable_pings --input_path /{location_of_data_folder}/ --save_path /{location_of_save_data_folder}/ -c dna_r9.4.1_450bps_hac.cfg -x 'auto' --recursive --num_callers 4 -- gpu_runners_per_device 8 --

chunks_per_runner 1024 --chunk_size 1000

The process generates fastq data files and places all reads with a minimum quality into a folder named “passed”.

7.2 Data analysis

To store and analyze the basecalled fastq nanopore read sequences obtained by sequencers from Oxford Nanopore Technologies (MinION, PromethION, GridION, Flongle) we use the software Geneious Prime v.21.1.1 or newer.

We developed an automated workflow that combines several steps including mapping reads to a reference database (in our case containing ca. 600 base pairs 5’ of the mitochondrial cytochrome oxidase I gene of insects, downloaded from the ‘Barcoding of Life Database’ BOLD), establishing a consensus sequence and running a BLAST search on a local copy of the GenBank database. The BLAST results which generally enable species identification of the sample are stored in a sub-folder.

1. Open the Geneious Prime software by clicking on the corresponding icon
2. In the left panel, go to your working folder and right-click to generate a new folder for storage and analysis of your new nanopore data files. We recommend to use a new folder for each run and within that folder a sub-folder for each barcoded sample. The nanopore fastq data files are stored in a folder on the computer system running the software ‘MinKNOW’ (which enables performing nanopore sequencing on the MinION as mentioned above) with a name similar to ‘passed’ and have the extension ‘.fastq’. For each barcoded sample, the corresponding fastq files have to be copied into the newly established data sub-folder in Geneious, for example, via drag and drop.
3. Select the desired number of read packets (by default, 4000 reads per packet), ideally ca. 200’000 in total, by activating them in the right-hand window, then select “Workflows – LB_MM2_PcRefSeq_NrSeq_ret30_210212” from the Geneious Prime menu. If that workflow (incl. in Appendix) does not appear on the menu you have to import it first.
4. In the new pop-up window, select the proper reference database – default is the custom made 60k entry database (RefDB_BOLD59kGBCocc755_60625Seq_211213.fasta; incl. in Appendix) extracted from BOLD and modified to exclude duplicates and to maintain a 97% minimal distance between branches. Upon confirming with ‘ok’ the workflow will perform the following steps using the parameters indicated below:

   a) Mapper: Minimap2 v.2.17 with the following parameters: Dissolve contigs

and re-assemble selected; reference sequence: RefDB_BOLD59kGBCocc755_60625Seq_211213; data type: Oxford Nanopore; include secondary alignments: maximum secondary aligments per read: 5; minimum secondary to primary alignment score ratio: 0.8; no trimming (remove existing trim regions from sequences); and under advanced options, an additional command line option: -t 8. Also, in the results panel, select 'Save assemply report', 'Save in sub-folder' and 'Save contigs'.

b) Sort Documents: Field to sort by: % of Ref Seq; select 'Reverse Sort' and 'After sorting, only keep at most 30 documents.

c) Mask Alignment: Eliminates alignment columns with <35% entries; in the Options panel, select 'Expose no options; in the Results panel, select 'Save a copy with sites stripped; in the What to mask or strip panel, select 'Sites containing: Gaps (%) 35%; in the All Operation Options panel, select 'Don't append 'Stripped' to sequence names.

d) Save Documents: Select 'Save these documents as output from workflow', 'Save in subfolder called: {Folder Name}; 'Select these documents when the operation completes, and 'Continue'.

e) Generate Consensus Sequence: Establishes a consensus sequence with a 0% majority threshold. Uses the following parameters: In the Options panel, select 'Expose no options; in the All Operation Options panel, select 'Threshold: 0% - Majority', 'Ignore Gaps', 'Assign Quality Total', 'Trim to reference sequence', and 'Append text to name of alignment consensus sequence'.

f) Save Documents: Select 'Save these documents as output from workflow', 'Save in subfolder called: {Folder Name}; 'Select these documents when the operation completes, and 'Continue'.

g) BLAST: Performs a BLAST search for the 'contig consensus sequence ' on a local GenBank database copy using the program 'Megablast', places results into a 'Hit table', retrieving 'Matching regions with annotations', and saves the top 10 best hits in a new folder. Uses the following parameters: In the Options panel, select 'Expose no options; in the All Operation Options panel, select 'Nucleotide Query Option', Database 'Nucleotide collection (nr/nt), Program: 'Megablast', Results: 'Hit table', Retrieve 'Matching region with annotations', Macimum Hits '10'. Also selected is 'Low Complexity Filter' and 'Mask for lookup table', with other parameters being default values.

h) Save Documents: Select 'Save these documents as output from workflow', 'Save in subfolder called: {Folder Name}; and 'Branch from 2 Operations Ago'.

i) Sort Documents: In the Options panel, select 'Expose no options; In the Options panel, select Field to sort by: 'Bit-Score', and select 'After sorting, only keep at most 30 documents.

The BLAST results in the new folder are sorted according to the highest Bit-Score for the hit which in most cases will present the highest % Pairwise Identity and the lowest probability value (E-Value) on top of the list. In addition, Geneious Prime adds other information for proper qualification of the BLAST hits.

Generally, the results obtained with this workflow provide '% Pairwise Identity' hits of **>99%** and hence strong evidence for an unambiguous species identification of the sample for which the consensus sequence was established.

7.3 Additional Resources

Geneious Prime: Training videos and other resources for all steps outlined above for the software Geneious Prime are available on the Geneious homepage: Resources | Geneious Prime
Barcoding of Life Database: The Barcode of life systems page (http://www.boldsystems.org/) is an excellent resource for barcoding based identifications and it allows searching their database with the proper marker sequence (e.g., COI for insects and ITS for fungi; see detailed instructions below).

8 Barcode-based identification on the NCBI GenBank Database

As an alternative to the identification using local BLAST implemented in the Geneious Prime workflow, the consensus sequence may also be BLASTed directly on the GenBank Database of NCBI:

1. Open the BLAST homepage of NCBI (https://blast.ncbi.nlm.nih.gov/Blast.cgi) and choose the web BLAST option 'Nucleotide BLAST'.
2. Paste your consensus sequence into the window 'Enter Query Sequence'
3. Check that the following parameters are chosen: *a.* Under 'Choose Search Set', 'Database': Choose 'Standard', and 'Nucleotide collection (nr/nt)' in the dropdown menu; leave the other fields empty. *b.* Under 'Program selection', 'Optimize for': Choose 'Highly similar sequences (megablast) *c.* Select 'Show results in a new window' before you select 'BLAST' at the bottom left of the web page *d.* It may take a short while before GenBank returns the BLAST results in a new window:
4. Criteria for correct species allocation are, among others: *a.* The top >10 entries are the same species name, however, there may be synonymous names for many insects. *b.* The identity of the query sequence with the GenBank entry (column 'Ident') is >97% *c.* The coverage region of the alignment between your consensus sequence and the GenBank entry is >80% (at least 300bp) *d.* The E-Value is very close to 0

> Note: GenBank does contain erroneous entries that may, for example, originate from an error in allocating the sample to the correct species based on morphological characters. Such cases can be identified easily if there is only a single such entry in a long list of identical species names.

9 Barcode-based identification on the Barcode of Life Database

In order to identify what species your consensus, full-length COI sequence originates from it is necessary to utilize reference databases. One example for such reference database is the Barcode of Life Database (BOLD). This database includes a comprehensive set of COI sequence data that has been collected by individuals and organizations across the globe and is constantly being updated with new data.

1. Start by navigating to the BOLD Systems webpage (http://www.boldsystems.org/) and select the “Identification” tab at the top of the webpage.
2. Using the default settings, select the “Animal Identification (COI)” tab (for arthropod identification) and the “Species Level Barcode Records” database. Paste the consensus sequence obtained from the sample into the search box at the bottom of the page.
3. The browser will eventually update and return the results of the search, revealing the records contained in the database that yield the closest match in terms of sequence similarity. The result screen contains a lot of information that may be explored to establish a confident identification for the query sequence. Often the search results will all originate from a single species allowing an unambiguous identification to be made for the sample. In some cases, two or more species are returned, prompting BOLD to display the message “A species match could not be made, the queried specimen is likely to be one of the following”. This may, for example, happen when highly similar reference sequences were entered with different names (synonyms) for the same species.

10 Barcode-based identification on the EPPO-Q-Bank Database

1. Open the EPPO-Q-Bank homepage (https://qbank.eppo.int/blast?db=arthropods), check that ‘Arthropods’ are selected and paste your consensus sequence into the window ‘Paste sequence to align:’ and select ‘Start alignment’
2. EPPO-Q-Bank will return its results in the lower part of the same window:
3. The percent identity of your query sequence to the best match entries in the EPPO-Q-Bank Database is indicated in the column ‘Similarity %’, the percent

overlap of both sequences in the column ‘Overlap %’. If both values are close to 100% then the species allocation is reliable.

Note: The three databases mentioned above are basically independent, although there is much overlap between GenBank and BOLD. This means that not all species are represented in all databases but rather in only one or two of them. Furthermore, the set of individual references for a species is mostly different in each database. Therefore, BLAST results will generally be different among the databases.

MATERIALS & EQUIPMENT

The sections below report all the equipment and materials required to apply this protocol. N.B. Batch numbers of kits used must be recorded.

Water
General use: Double-distilled water, preferably from a Milli-Q ultraclean water device
PCR procedures: Sterile, DNase-, RNase- and Protease-free water e.g. Fisher Scientific DNA free water, product code: BPE2470-1.

Water DNA Grade DNASE Protease free **Fisher Scientific Catalog #BP24701**

Solutions, standards and reference materials
Solutions: All solutions should be of molecular grade purity and only be used to the expiry date indicated on the package. Repeated freeze-thaw cycles should be avoided. Pipetting should be performed with utmost care using filter tips to avoid contamination. Where appropriate aliquots may be used to minimize contamination.

Standards: All standards should only be used to the expiry date indicated on the package. Repeated freeze-thaw cycles should be avoided. Pipetting should be performed with utmost care using filter tips to avoid contamination.

Reference materials: The reference library may be established with any collection of nucleic acid sequences that are useful to discriminate among the taxa of interest. For example, in the case of insects, the reference library is composed of all insect standard barcode entries (a 648 base-pair region of the mitochondrial cytochrome c oxidase 1 gene (“CO1” or “COI”) downloaded from the ‘Barcoding of Life Database’ (http://www.barcodinglife.org/; downloaded in May 2021), with identical entries removed and a minimum difference between tree tips of 3%. This was done with the utility “Dedupe” from BBTools (BBTools - DOE Joint Genome Institute; as implemented in Geneious Prime) to remove duplicate entries and all sequences with a similarity of >97% among each other.

The present SOP was successfully used, partly with minor variations, on a total of 67 samples covering 14 insect families and 26 species.

Commercial kits
Nucleic acid extraction: The method described here uses the NEB Monarch® Genomic DNA Purification Kit (New England Biolabs Catalog #T3010S) and the Qiagen DNeasy® PowerClean® Cleanup Kit (order nr. 12877-50), as described in the Section ‘Steps’. However, the process also worked with the following commercial kits (data not shown): ‘GenElute™ Mammalian Genomic DNA Miniprep Kit’ (Sigma-

Aldrich Chemie GmbH, Buchs, Switzerland; Product code G1N350) and the 'DNeasy Blood & Tissue Kit' (QIAGEN AG, Basel, Switzerland; Product code 69506). The workflow was also successfully used starting with RNA extraction. This has the added benefit of enabling to look for potential arboviruses in the data. However, this requires that more data are collected. A reverse transcription step is required if using an RNA extraction kit. For example, using the GenElute™ Total RNA Purification Kit from Sigma-Aldrich (Merck, Sigma-Aldrich Chemie GmbH, Buchs, Switzerland; Product code: RNB100), the following cDNA production kit was successfully used: LunaScript® RT SuperMix Kit (Bioconcept AG, Allschwil, Switzerland; Product code NEB E3010S). Also, if RNA is extracted it may be beneficial to omit the DNAse step to maximize the yield of nucleic acids.

Monarch® Genomic DNA Purification Kit **New England Biolabs Catalog #T3010S**

DNeasy PowerClean CleanUp Kit **Qiagen Catalog #12877-50**

GenElute™ Mammalian Genomic DNA Miniprep Kit **Sigma Aldrich Catalog #G1N350**

DNeasy Blood & Tissue Kits **Qiagen Catalog #69506**

GenElute™ Total RNA Purification Kit **Sigma Aldrich Catalog #RNB100**

LunaScript® RT SuperMix Kit **New England Biolabs Catalog #E3010S**

Whole Genome Amplification (WGA): GenomePlex® Complete Whole Genome Amplification Kit WGA2 (Sigma-Aldrich Chemie GmbH, Buchs, Switzerland; Product code WGA2-50RXN)

GenomePlex® GGA Kit zur Gesamtgenom-Amplifikation **Sigma Aldrich Catalog #WGA2-50RXN**

cDNA Production: LunaScript® RT SuperMix Kit (Bioconcept AG, Allschwil, Switzerland; Product code NEB E3010S)

LunaScript® RT SuperMix Kit **New England Biolabs Catalog #E3010S**

Nanopore Sequencing by MinION: Amplicons by Ligation (SQK-LSK109) with native barcode ligation: Ligation Sequencing Kit SQK-LSK109 with Native Barcoding Expansion 1-12 Kit (EXP-NBD104).

Native Barcoding Expansion 1-12 (PCR-free) **Oxford Nanopore Technologies Catalog #EXP-NBD104**

NEBNext® Companion Module for Oxford Nanopore Technologies® Ligation Sequencing (Bioconcept AG, Allschwil, Switzerland; Product code NEB E7180S)

NEBNext® Companion Module for Oxford Nanopore Technologies® Ligation Sequencing **New England Biolabs Catalog #E7180S**

Reaction Cleanup: MinElute Reaction Cleanup Kit of Qiagen (QIAGEN AG, Basel, Switzerland; Product code 28206)

MinElute Reaction Cleanup Kit **Qiagen Catalog #28206**

Plastic ware and other disposable material

It is essential that all plastic-ware is sterile before use.

A	B	C	D
Item	Detail	Example Supplier	Product code / Remarks
Pipette tips (filtered)	10, 100, 200 & 1000µl	Fisher Scientific	
PCR tubes	single, strip or 96-well	Fisher Scientific	
Eppendorf tubes	0.2, 0.5, 1.5 ml	Fisher Scientific	lowBind
Qubit assay tubes		Invitrogen (Fisher Scientific)	lowBind
SeqStudio Microplates	MicroAmp Optical 96 well<br><br>Reaction Plate	Applied Biosystems by life technologies (Fisher Scientific)	
Retsch Collection Microtubes	2ml	Qiagen	Qiagen Collection Microtubes (cat. no. 19560); Qiagen Collection Microtube Caps (cat. no. 19566)
Retsch Beads (stainless steel grinding beads)	3mm	Fisher Scientific	11758414

Equipment
The following items of equipment are required to undertake the analysis. Several alternative suppliers/models are available for each item. These must be shown to be appropriate before use.

A	B	C	D
Item	Detail	Example supplier	Product code / Remarks
Precision pipettes	1-1000µl	Fisher Scientific	
Bench top vortex		Labnet	VX-100
Thermocycler	SensoQuest	Witec	
Thermal mixer	to hold 1.5 ml tubes	Eppendorf	5355

DNA quantifier - photospectrometric	Accurate to +/- 1 ng	Witec (Thermo Scientific)	Nanodrop ONE
DNA quantifier - fluorometric		Invitrogen (Fisher Scientific)	QuBit 3
Microcentrifuge	to hold 1.5 ml tubes	Eppendorf	5452
Desktop centrifuge with microwell plate carrier		Sigma	4-15C
Power supply for electrophoresis		BioRad	1000/500
Electrophoresis equipment (trays, combs)		BioRad	
UV documentation system		Witec	E-Box
Vacuum Pump for microwell plate 96 cleanup steps	≥20 inHG vacuum	Millipore	
Sequencing device	MinION Mk1B	Oxford Nanopore Technologies ONT	
Sequencing flowcell	R.9.4.1	Oxford Nanopore Technologies ONT	
Laminar flow hood	With UV light	SKAN	MonMouth VFLT1000
Tissue Homogenizer with Adaptors	TissueLyser II	Qiagen	
Magnet	MagJET Separation	Thermo Scientific™ (Thermo Fisher Scientific)	15265126
Tube rotator (HULA mixer)	RotoFlex® Plus bench top tube rotator	Merck (Sigma-Aldrich)	Z740290

Other materials
Disposable plastic gloves, sterile dissection equipment.

Electronic files / computer software
The software Geneious Prime (Biomatters; https://www.geneious.com/prime/) v. 21.1.1. or above with a single user license.
Internet access is required to utilize NCBI's BLAST (BLAST: Basic Local Alignment Search Tool (nih.gov)). Alternatively, a local installation of a BLAST database is required (may be performed with assistance from Geneious Prime after downloading the necessary nt files from the GenBank site).

For hazard information and safety warnings, please refer to the SDS (Safety Data Sheet).

All protocol steps using commercial kits generally follow exactly the recommendations of the suppliers, omitting some of the more detailed comments. Deviations from the supplier's protocols are clearly indicated.

This workflow performed successfully with tissue amounts corresponding to a single adult Thrips to pieces of max. 2 mg of, e.g., Tephritid larvae. If the weight of your insect sample is less than ca. 2 mg (e.g., an adult thrips or a small Drosophila species such as Drosophila suzukii) then use the entire sample. If the sample is larger (such as a Tephritid larva) cut off a small tissue sample of no more than 2 mg.

> It is essential to wear disposable plastic gloves during all laboratory procedures and to use pipette tips that are sterile and fitted with filters.

1 Sample preparation and DNA Extraction - 1.1 Tissue Disruption

1 Materials:

Disrupt tissue samples on the Retsch Mixer Mill TissueLyser II (Qiagen), using the Monarch® Genomic DNA Purification Kit (New England Biolabs NEB #T3010) and Qiagen Collection Microtubes (racked) and Collection Microtube Caps (cat. nos. 19560 and 19566 respectively).

Collection Microtubes (racked 10 x 96) **Qiagen Catalog #19560**

Collection Microtube Caps (120 x 8) **Qiagen Catalog #19566**

Notes before Starting:

- Add ethanol (≥ [M] **95 %**) to the Monarch gDNA Wash Buffer concentrate as indicated on the bottle label.
- Set a thermal mixer (e.g. ThermoMixer® or similar device), or a heating block to **56 °C** for sample lysis.
- Set a heating block to **60 °C** . Preheat the appropriate volume of elution buffer

to 60 °C (35 µL – 100 µL per sample). Confirm the temperature, as temperatures are often lower than indicated on the device.

2 All samples should be stored frozen at -20 °C or stored in [M] 70 % EtOH (few days at Room temperature or at 4 °C in the refrigerator) until processed. Samples can be stored frozen indefinitely. Use sterile dissection equipment where appropriate.

3

Add 200 µL Tissue Lysis Buffer and 10 µL Proteinase K to each sample.

4 Add one stainless steel ball 3 mm per sample.

5 Place max. 2 mg sample into a Collection Microtube (Qiagen).

6 6m

Disrupt tissue on the TissueLyser II for **2x** 00:03:00 at 25 Hz, turning plate after the first period. Briefly centrifuge once done.

Deviation from supplier's protocol.

7 30m

Incubate at 56 °C for 00:30:00 in a thermal mixer with agitation at full speed (1400 rpm) .

8 3m

Centrifuge for 00:03:00 at maximum speed (> 12.000 x g) to pellet debris. Transfer the supernatant to a fresh microfuge tube.

9 5m

Add **3 µL RNase A** to the lysate, vortex thoroughly and incubate for a minimum of **00:05:00** at **56 °C** with agitation at full speed.

1.2 DNA Binding and Elution

10 15s

Add **400 µL gDNA Binding Buffer** to the sample and mix thoroughly by pulse-vortexing for **00:00:05** - **00:00:10** .

11 4m

Transfer the lysate/binding buffer mix (~ **600 µL**) to a gDNA Purification Column pre-inserted into a collection tube, without touching the upper column area. Close the cap and centrifuge: first for **00:03:00** at **1.000 x g** to bind gDNA (no need to empty the collection tubes or remove from centrifuge) and then for **00:01:00** at maximum speed (> **12.000 x g**) to clear the membrane. Discard the flow-through and the collection tube.

12 1m

Transfer column to a new collection tube and add **500 µL gDNA Wash Buffer** . Close the cap and invert a few times, so that the wash buffer reaches the cap. Centrifuge immediately for **00:01:00** at maximum speed (**12.000 x g**), and discard the flow through.

13 1m

Reinsert the column into the collection tube. Add **500 µL gDNA Wash Buffer** and close the cap. Centrifuge immediately for **00:01:00** at maximum speed (>

12.000 x g), then discard the collection tube and flow through.

14 1m

Place the gDNA Purification Column in a DNase-free 1.5 ml microfuge tube (not included). Add **50 µL preheated (60°C) gDNA Elution Buffer** , close the cap and incubate at **Room temperature** for **00:01:00** .

15 1m

Centrifuge for **00:01:00** at maximum speed (> **12.000 x g**) to elute the gDNA.

2 Reaction Cleanup - 2.1 Cleanup of eluted DNA 20m

16 Materials:

The reaction cleanup is performed using the DNeasy® PowerClean® Cleanup Kit (Qiagen order nr. 12877-50) according to the manufacturer's recommendations.

DNeasy PowerClean CleanUp Kit **Qiagen Catalog #12877-50**

Notes before Starting:

- Shake to mix solution SB.
- If Solution SL has precipitated, heat at **60 °C** , gently inverting the tube periodically until the precipitate has dissolved. Solution SL may be used while still warm.

17

Add **100 µL double-distilled (PCR grade) water** to the **50 µL of eluted DNA** .

18 Transfer **150 µL diluted DNA eluate** to a clean 2 ml collection tube (provided).

19

Add **70 µL Solution CU** to the DNA. Gently invert 5 times.

20

Add **20 µL Solution SL** and invert 5 times.

21 5m

Add **85 µL Solution AA** and invert 5 times. Incubate at **4 °C** (e.g., in a refrigerator) for **00:05:00** .

22 1m

Centrifuge at **10000 x g, Room temperature , 00:01:00** .

23 Transfer supernatant to clean 2 ml collection tube (provided), do not disturb pellet.

24 5m

Add **70 µL Solution IRS** and invert 5 times. Incubate at **4 °C** for **00:05:00** .

25 1m

Centrifuge at **10000 x g, Room temperature , 00:01:00** .

26 Transfer supernatant to clean 2 ml collection tube (provided), do not disturb pellet.

27 5s

Add **800 µL Solution SB** and vortex for **00:00:05** .

28 1m

Load **600 µL** onto an MB Spin Column and centrifuge at **10000 x g, Room temperature , 00:01:00** . Discard flow through.

29 1m

Add the remaining **600 µL supernatant** to the MB Spin Column and centrifuge at **10000 x g, Room temperature , 00:01:00** .

30 30s

Add **500 µL Solution CB** to the MB Spin Column and centrifuge at **10000 x g, Room temperature , 00:00:30** . Discard flow through.

31 1m

Centrifuge the MB Spin Column at **13000 x g, Room temperature , 00:01:00** .

32 Carefully place the MB Spin Column in new 2 ml collection tube (provided). Avoid splashing any Solution CB onto the MB Spin Column.

33 1m

Add **50 µL Solution EB** to the center of the white filter membrane. Incubate for **00:01:00** at **Room temperature** .

34 Centrifuge at **10000 x g, Room temperature , 00:00:30** . 30s

35 Discard the MB Spin Column. Continue with WGA or store cleaned DNA frozen at **-20 °C** .

2.2 DNA quantification

36

DNA extracted with a commercial kit or after clean-up may be quantified to assess the extraction process and enable normalisation of DNA concentration. One common method is to use a Qubit 3 fluorometer or, alternatively, a Nanodrop ND 1000 spectrophotometer. DNA should be diluted to 10-50ng/µl using DNA-free water. Negative controls should read ~0 ng/µl.

Controls:
A negative extraction control (with no tissue) should be run in parallel with all batches of sample extraction and quantified alongside all tissue extractions

3 Whole Genome Amplification (WGA)

37 Materials:

We use the GenomePlex® Complete Whole Genome Amplification Kit WGA2 (Sigma-Aldrich Chemie GmbH, Buchs, Switzerland; Product code WGA2-50RXN).

GenomePlex® GGA Kit zur Gesamtgenom-Amplifikation **Sigma Aldrich Catalog #WGA2-50RXN**

Procedure for whole genome amplification using the Sigma GenomePlex® Complete Whole Genome Amplification Kit WGA2:

3.1 WGA Step 1: Fragmentation 34m

38 30m

Run Thermocycler program (Program: incubation at **95 °C** , runs **00:30:00**). (To assure the Thermocycler is ready when needed.)

39

Use DNA/cDNA sample: Transfer **10 µL DNA (≥ 10 ng)** of section 2.2/step 19 **into new 8-Strip Microtubes**.

40

Add **1 µL Fragmentation Buffer** to each DNA tube of previous step.

41 Heat for 00:04:00 @ 95 °C in Thermocycler. **Immediately cool** On ice . 4m

Alternatively, a tabletop Mini Cooler may be used.

3.2 WGA Step 2: Library Preparation 2m

42

Add **2 µL Library Preparation Buffer (green)** to DNA of previous step.

43

Add **1 µL Library Stabilization Solution (yellow)** to DNA of previous step. Vortex and centrifuge.

44 Heat for 00:02:00 @ 95 °C in Thermocycler. **Immediately cool** On ice . 2m

45

Add **1 µL Library Preparation Enzyme (red)** to DNA of previous step. Vortex and centrifuge.

46 1h 5m

Run Thermocycler program with WGA Library Prep Rxn.

Program:

incubation at 16 °C , runs 00:20:00 ;

incubation at 24 °C , runs 00:20:00 ;

incubation at 37 °C , runs 00:20:00 ;

incubation at 75 °C , runs 00:05:00 ;

cool to and hold at 4 °C

3.3 WGA Step 3: Amplification 30m

47

Add **48 µL MH2O** to each reaction tube of previous step (WGA Library Prep Rxn).

48

Add **7.5 µL Amplification Master Mix** to each reaction tube of previous step.

49

Add **5 µL WGA DNA Polymerase** to each reaction tube of previous step. Vortex and centrifuge.

50 Run Thermocycler program. 8m 15s

Program:

Initial incubation: **00:03:00** at **95 °C** ;

17 cycles of **00:00:15** at **94 °C** ; **00:05:00** at **65 °C** ;

cool to and hold **4 °C**

Store short term **4 °C** , long term **-20 °C** .

51 30m

OPTIONAL: Check on gel: Run **5 µL** on 1.4% TBE gel, **4 µL marker** , **4 µL loading dye** at 70 V/cm for **00:30:00** .

3.4 WGA Step 4: Reaction Cleanup 6m

52

Prepare MinElute column setup on 2ml collection tube.

53 Add **300 µL Buffer ERC** to **75 µL WGA product** .

54 Load **375 µL of mixture** to column setup.

55 1m

Centifuge the sample of last step for **00:01:00** .

Discard flow-through, re-assemble.

56

Add **750 µL Buffer PE** to column setup.

57 1m

Centifuge the sample of last step for **00:01:00** .

Discard flow-through, re-assemble.

58 2m

Centrifuge the sample of last step for **00:02:00** .

Place MinElute column in **NEW 1.5ml Eppendorf tube**.

59

Add **10 µL Elution Buffer** to centre of MinElute Column.

Deviation from supplier's protocol.

60 1m

Incubate for **00:01:00** @ **Room temperature** .

61 1m

Centrifuge for **00:01:00** .

> DNA quantification at this step may be advisable if the expected yield is below 10 ng.

4 WGA Product Check by Gel Electrophoresis

62 Gel electrophoresis of DNA in an agarose gel is a standard technique in molecular biology, but equipment, reagents, staining and visualization varies considerably between laboratories, and according to local health & safety controls. Therefore, this SOP suggests general conditions that need to be adapted to each laboratory.

4.1 Make a 1.2% TBE agarose gel (1xTBE pH: 9.0) containing 0.0001% Ethidium Bromide*)

32m

63

Place **80 mL 1xTBE-Buffer** + **1 g Agarose** in a 500ml Erlenmeyer flask.

64 2m

Heat in microwave at max intensity for **00:02:00** with intermittent interruption for shaking (take care not to overheat).

65

Add **8 µL Ethidium Bromide*)** and cast the gel.

> *) Ethidium Bromide is a carcinogenic chemical, use nitrile gloves and consult the security regulations.

66 Wait 00:30:00 at Room temperature or store in the fridge at 4 °C . 30m

4.2 Gel loading and running 30m

67

Prepare Size Standard (e.g. Thermo Scientific™ GeneRuler DNA Ladder Mix, ready-to-use; Order Nr. 10181070) and samples (whole genome amplification products) for gel loading by mixing **3 µL of each sample and of the Size Standard** with **3 µL of Loading buffer** to be prepared as follows:

Loading Buffer Preparation for PCR Amplification Product Electrophoresis (10ml):

67.1

Add **1.5 g Ficoll 400** to **10 mL 1xTBE** , adjust pH to pH**9.0** .

67.2

Add **5 mg Bromophenol Blue** (adjust amount visually, may be too high).

68

Carefully pipet the **6 µL loading mix** into individual wells of the gel, beginning with the Size Standard at the leftmost well.

69 Run at 70V for approximately 00:30:00 (depending on size of gel), ensuring the DNA does not run off the gel. 30m

4.3 Visualization

70 Visualize your DNA fragments in UV light (with appropriate safety precautions); if the

WGA reaction has been successful it shows as a smear of approximately 400 - 1000 base pairs in length. Your negative controls should not contain bands.

71

> Note: If no WGA amplification signal is obtained after several attempts it may be advisable to run a positive control using a previously successful PCR. In rare cases more DNA extract may be needed. Alternatively, there may be inhibitors for the PCR in the crude extract, such as in aphids, where the high sugar content inhibits PCR. In such cases, the crude extract needs to be cleaned up with a commercial kit such as the Sigma 'GenElute™ Mammalian Genomic DNA Miniprep Kit.

4.4 Recording

72 Keep a permanent record of your gel (electronic and/or hard copy) as proof that the WGA reaction was successful and contaminant free.

5 Sequencing Library Preparation

73

> Protocols of ONT for library preparation, priming and loading change frequently. Please check the ONT website for updates.

Materials:

The library for nanopore sequencing is produced with the Ligation Sequencing Kit SQK-LSK109 of Oxford Nanopore Technologies for sequencing on the flowcell type R.9.4.1 (flowcell ID: FLO-Min106D), following the manufacturer's recommendations with some minor modifications.

Ligation Sequencing Kit **Oxford Nanopore Technologies Catalog #SQK-LSK109**

5.1 Library Preparation Step 1: DNA End-Prep 1h

74

Transfer 120 ng total DNA of section 3 **into new 8-strip Microtubes**.

75

Add MH2O to total **54 µL** .

76

Add **3.5 µL Ultra II End-prep reaction buffer** .

77

Add **3 µL Ultra II End-prep enzyme mix** .

78 Mix by flicking, spin down.

79 50m

RUN Thermocycler program.

Program:
incubate **00:30:00** at **20 °C** / **00:20:00** at **65 °C** ,
transfer contents to 1.5ml Eppendorf tube.

80

Add **60 µL AMPure XP beads** (resuspended). Mix by flicking tube.

81 10m

Incubate for **00:10:00** @ **Room temperature** on HULA mixer.

82

Spin and pellet on magnet until clear. Pipette off supernatant, keep on magnet.

83

Wash beads on magnet with **200 µL 70% EtOH (fresh)** . Pipette off supernatant, do not disturb pellet.

84

Wash beads on magnet with **200 µL 70% EtOH (fresh)** . Pipette off supernatant, do not disturb pellet.

85

On magnet, pipette off residual EtOH.

86 Dry for **00:00:30** . 30s

87 Resuspend in **61 µL water (nucleasefree)** .

88 5m

Incubate for **00:05:00** at **Room temperature**

89 Pellet on magnet until clear.

90 Collect **61 µL** eluate. May store at **4 °C** **Overnight** . 5m

91

OPTIONAL: Quantify **1 µL** of product on QuBit. Note DNA concentration (ng/µl).

5.2 Library Preparation Step 2: Native Barcode Ligation 17m 30s

92

Add **22.5 µL** of eluted DNA of Endrepair product into new 1.5 ml Eppendorf tube; mix by pipetting. Use total **100 fmol** - **200 fmol** (=ca. 35-60 ng).

93

Add **2.5 µL Native Barcode** to each reaction tube of previous step. **Note Barcode Numbers!**

94

Add **25 µL Blunt/TA Ligase Master Mix** to each reaction tube of previous step; mix by pipetting.

95 10m

Incubate for **00:10:00** @ **Room temperature** .

96

Add **50 µL AMPure XP beads** (resuspended). Mix by flicking.

97 5m

Incubate for **00:05:00** @ **Room temperature** on HULA mixer. Spin down.

98

Pellet on magnet until clear. Pipette off supernatant, keep on magnet.

99

Wash beads on magnet with **200 µL 70% EtOH (fresh)** . Pipette off supernatant, do not disturb pellet.

100

Wash beads on magnet with **200 µL 70% EtOH (fresh)** . Pipette off supernatant, do not disturb pellet.

101

Remove residual by spin on magnet. Pipette off residual EtOH.

102 Dry for **00:00:30** . 30s

103 Resuspend in **26 µL water (nucleasefree)** .

104 2m

Incubate for **00:02:00** @ **Room temperature** .

105 Pellet on magnet until clear.

106 Collect **26 µL eluate** and transfer to 1.5ml Eppendorf tube.

107

MUST DO: Quantify **1 µL** of product on QuBit. Note DNA concentration (ng/µl).

108 Pool **equimolar amounts** of each barcoded sample to 1.5ml Eppendorf tube (to 100-200 fmol total (=ca. 60 ng)).

DO NOT multiply low concentration samples linearly! @10x lower concentration use max 5x more DNA!

109 Dilute single pooled Barcode ligation product to **65 µL** .

5.3 Library Preparation Step 3: Adaptor Ligation and Clean-up 35m 30s

110 Use **60 µL single pooled barcode ligation product** .

111

Add **25 µL Ligation Buffer (LNB)** .

112

Add **10 µL NEBNext Quick T4 DNA Ligase** .

113

Add **5 µL Adapter Mix (AMII for multiplex)** . Mix by flicking, spin down.

114 10m

Incubate for **00:10:00** @ **Room temperature** .

115

Add **40 µL AMPure XP beads (resuspended)** . Mix by flicking

116 15m

Incubate for **00:15:00** @ **Room temperature** on HULA mixer. Spin down.

117

Pellet on magnet until clear. Pipette off supernatant, keep on magnet.

118 3m

Wash beads with **250 µL Short Fragment Buffer (SFB)** . Wait **00:03:00** on Magnet. Resuspend by flicking, pellet, remove supernatant.

119 3m

Wash beads with **250 µL Short Fragment Buffer (SFB)** . Wait **00:03:00** on Magnet. Resuspend by flicking, pellet, remove supernatant.

120 Remove residual by spin.

121 Dry **00:00:30** . 30s

122 Resuspend in **15 µL Elution Buffer (EB)** .

123 10m

Incubate for **00:10:00** @ **37 °C** .

124 Pellet on magnet until clear.

125 Collect library (**15 µL**) from previous step into new Eppendorf tube.

126

Quantify **1 µL eluted DNA** on QuBit. Use appropriate amount for 16 ng of library for next step; dilute with EB buffer.

6 Nanopore Sequencing

127 Priming and Loading the Flowcell

6.1 Nanopore Sequencing Step 1: Priming and Loading the Flowcell 5m

128 Prepare Flowcell (perform QC on MinION). Record number of active pores.

129

Prepare the flow cell **priming mix**: Add **30 µL Flush Tether (FLT)** directly to Flush Buffer (FB) tube. Mix.

130 Load flow cell with **800 µL priming mix** via priming port. Spot on closed!!!

131 5m

Wait for **00:05:00** .

132 Prepare **Library** for loading: add **38 µL Sequencing Buffer (SQB)** to 1.5ml Eppendorf tube.

133 Add **26 µL Loading Beads (LB)** to 1.5ml Eppendorf tube (mixed immediately

before use).

134 Add **12 µL diluted DNA library** from section 5.3 (step 125) to 1.5 ml Eppendorf tube.

> We used up to 16 ng total DNA but obtained best results with 12 ng.

135 Load flow cell with **200 µL priming mix** via priming port. Spot on closed!!!

136 Add **75 µL Library** from step 134 via SpotON sample port. Add drop by drop!

137 Close priming port, SpotOn port, **perform sequencing** on a MinION (Flongle, GridION, Promethion) using protocols.io method https://www.protocols.io/view/starting-a-minion-sequencing-run-using-minknow-7q6hmze; make sure to use flow cell type LSK109 and barcode kit EXP-NBD104 (option now available).

6.2 Nanopore Sequencing Step 2: Flow Cell Storage 30m

138

Prepare wash mix: Add **20 µL Wash Solution A** to 1.5ml Eppendorf tube.

139

Add **380 µL Wash Solution B** to same 1.5ml Eppendorf tube. Vortex.

140 Open inlet port, add **400 µL Wash Mix** via priming port. Close priming port after loading (Spot on closed!!!).

141 30m

Wait for **00:30:00** @ **Room temperature** .

142 Add **500 µL Storage Buffer S** via priming port. Close priming port after adding (Spot on closed!!!).

143 Remove spare contents in flow cell. Aspirate **1000 µL AIR** from empty flow cell via trash removal port top left. Spot on closed!!!

144 Store in fridge.

7 Raw Data Processing and Analysis

145

For Raw Data Processing and Analysis, please see section "Guidelines".
