# Supplementary figures and images for: Next generation biosecurity: Towards genome based identification to prevent spread of agronomic pests and pathogens using nanopore sequencing
Source: PLoS One. 2022 Jul 25;17(7):e0270897. doi: 10.1371/journal.pone.0270897 (PMC9312391; doi:10.1371/journal.pone.0270897)

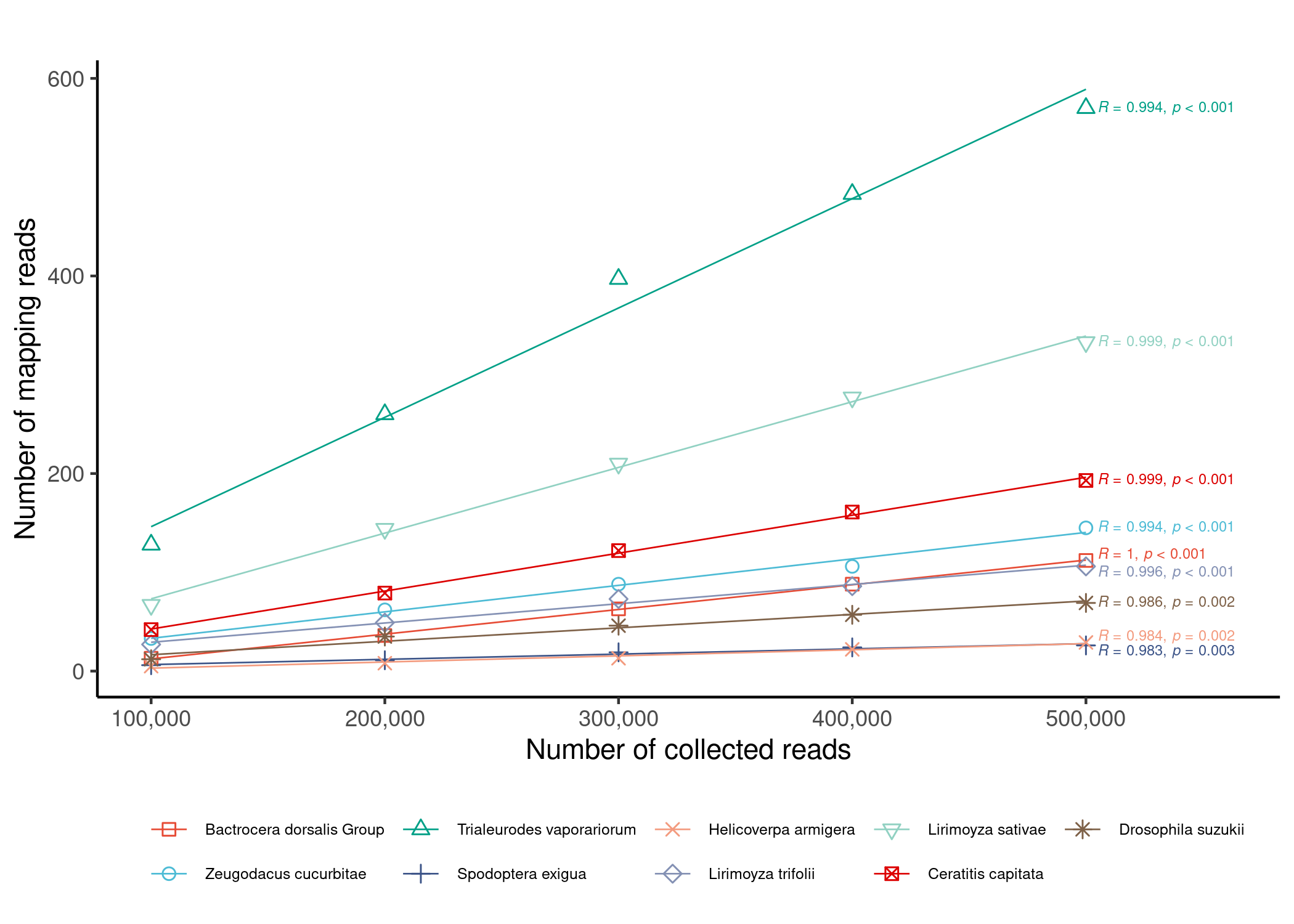

Supplement: S1 Fig — Included are samples for which at least 500’000 reads were sampled and more than four mapping reads were found: Bactrocera dorsalis, Ceratitis capitata, Drosophila suzukii, Helicoverpa armigera, Liriomyza sativae, Liriomyza trifolii, Spodoptera exigua, Trialeurodes vaporariorum, Zeugodacus cucurbitae. (TIF) [file pone.0270897.s007.tif]
