# Supplementary material for: Next generation biosecurity: Towards genome based identification to prevent spread of agronomic pests and pathogens using nanopore sequencing
Source: PLoS One. 2022 Jul 25;17(7):e0270897. doi: 10.1371/journal.pone.0270897 (PMC9312391; doi:10.1371/journal.pone.0270897)
Supplement: Attachment — Submitted filename: Respones to Reviewers.docx [file pone.0270897.s008.docx]

Response to Reviewers and the Editor

1. Please ensure that your manuscript meets PLOS ONE's style requirements, including those for file naming. The PLOS ONE style templates can be found at 
<https://journals.plos.org/plosone/s/file?id=wjVg/PLOSOne_formatting_sample_main_body.pdf> and 
<https://journals.plos.org/plosone/s/file?id=ba62/PLOSOne_formatting_sample_title_authors_affiliations.pdf>

We’ve revised the manuscript according to these two guidelines.

2. We noted in your submission details that a portion of your manuscript may have been presented or published elsewhere. [This submission is part of the protocols.io/PLoS one collaboration. The corresponding protocols.io document is available here:
dx.doi.org/10.17504/protocols.io.bx7nprme
The materials & methods section is very similar to the one on protocols.io but the intro/results/discussion are only presented here]  Please clarify whether this publication was peer-reviewed and formally published. If this work was previously peer-reviewed and published, in the cover letter please provide the reason that this work does not constitute dual publication and should be included in the current manuscript.

No, this manuscript has not been published or submitted elsewhere. The protocols.io has not been peer reviewed as to our knowledge, none of the protocols on protocols.io are. As we are informed, this is how the protocols.io/PLoS one collaboration is intended. To publish a protocol without peer review and add some more insights, applying the presented protocol to a specific use case and adding Introduction, Results and Discussion to a peer-reviewed PLoS one article

3. We note that you have stated that you will provide repository information for your data at acceptance. Should your manuscript be accepted for publication, we will hold it until you provide the relevant accession numbers or DOIs necessary to access your data. If you wish to make changes to your Data Availability statement, please describe these changes in your cover letter and we will update your Data Availability statement to reflect the information you provide.

The data is on SRA under PRJNA783774 and ready to be released as soon as the MS is accepted.

4. We note that you have included the phrase “data not shown” in your manuscript. Unfortunately, this does not meet our data sharing requirements. PLOS does not permit references to inaccessible data. We require that authors provide all relevant data within the paper, Supporting Information files, or in an acceptable, public repository. Please add a citation to support this phrase or upload the data that corresponds with these findings to a stable repository (such as Figshare or Dryad) and provide and URLs, DOIs, or accession numbers that may be used to access these data. Or, if the data are not a core part of the research being presented in your study, we ask that you remove the phrase that refers to these data.

We have deleted the “data not shown” and have revised the statement in the results section in a way that no results need to be shown.

Reviewers' comments:

Reviewer's Responses to Questions

**Comments to the Author**


1. Does the manuscript report a protocol which is of utility to the research community and adds value to the published literature?

Reviewer #1: Yes

Reviewer #2: Yes

 2. Has the protocol been described in sufficient detail?

Descriptions of methods and reagents contained in the step-by-step protocol should be reported in sufficient detail for another researcher to reproduce all experiments and analyses. The protocol should describe the appropriate controls, sample sizes and replication needed to ensure that the data are robust and reproducible.

Reviewer #1: Yes

Reviewer #2: Yes

 3. Does the protocol describe a validated method?

The manuscript must demonstrate that the protocol achieves its intended purpose: either by containing appropriate validation data, or referencing at least one original research article in which the protocol was used to generate data.

 Reviewer #1: Yes

Reviewer #2: Yes

4. If the manuscript contains new data, have the authors made this data fully available?

The [PLOS Data policy](http://www.plosone.org/static/policies.action#sharing) requires authors to make all data underlying the findings described in their manuscript fully available without restriction, with rare exception (please refer to the Data Availability Statement in the manuscript PDF file). The data should be provided as part of the manuscript or its supporting information, or deposited to a public repository. For example, in addition to summary statistics, the data points behind means, medians and variance measures should be available. If there are restrictions on publicly sharing data—e.g. participant privacy or use of data from a third party—those must be specified.

All the sequence data is already deposited at the SRA under PRJNA783774 and will be made available upon acceptance of the manuscript.

Reviewer #1: Yes

Reviewer #2: Yes

**5. Is the article presented in an intelligible fashion and written in standard English?**


PLOS ONE does not copyedit accepted manuscripts, so the language in submitted articles must be clear, correct, and unambiguous. Any typographical or grammatical errors should be corrected at revision, so please highlight any specific errors that need correcting in the box below.

 Reviewer #1: Yes

Reviewer #2: Yes

6. Review Comments to the Author

Please use the space provided to explain your answers to the questions above. You may also include additional comments for the author, including concerns about dual publication, research ethics, or publication ethics. (Please upload your review as an attachment if it exceeds 20,000 characters)

Reviewer #1: ID PONE-D-22-03204: “Next generation biosecurity: Towards genome based identification to prevent spread of agronomic pests and pathogens using nanopore sequencing” by Frey et al.

The manuscript reports a protocol that can be useful to the research community interested in the identification of agronomic pests. The traditional methods are based on classical barcoding (or metabarcoding) that targets one or a few genomic regions of one or a small group of species. The barcoding approach requires some prior knowledge of the target organisms, which sometimes is lacking. In this manuscript, the authors take the metagenomic approach by sequencing all the DNA of the sample, so any genomic region of any organism can be sequenced and, if there is enough genomic information in the repositories, identified. In consequence, the proposal is more flexible than the classical one based on barcoding. I reckon that in the future (with richer genomic repositories) methods like the one proposed here based on metagenomics will overcome (meta)barcoding methods.

The protocol is described in sufficient detail in the main text and the supplementary material. The method is validated using more than 60 artificial single-species libraries of insects and a few more of bacteria, fungi, and nematodes.

Despite being very positive about the manuscript, I don’t like how results are presented. I have a couple of suggestions that may be useful to the authors in the preparation of a new version of the manuscript.

Perhaps Figure 1 is unnecessary. It is just to be expected to find a highly significant linear relationship between the number of mapping reads and the number of collected reads. This information can be included in the text.

We agree with the reviewer that the data shown in Figure1 is somewhat trivial. We thus moved the Figure to Supplementary Figure1

On the contrary, I’ve found the description of sample identification too shallow (l. 231-237). The authors only provide summary statistics (Table 1 and 2) that show that the process of species identification is good, in general. However, this is not always the case, as there are 9 libraries in which the “assumed species” is different to the “species ID” (Supplementary Table 2: please clarify the meaning of these two columns). This criticism does not invalidate the method but is proof that the method is not perfect (as it happens with all DNA-based technologies for species identification). I encourage the authors to acknowledge the limitations of their method in the main text (in the results) and explain, if possible, the reason for the wrong or partial identification of some species (in the discussion).

The author is right that for 12 libraries the assumed species is different than the species ID. The assumed species stems a morphological “identification” or “best guess” by experienced layman working at the airport or by entomologists working at our institution. Some of the samples consist of larvae which are hard to distinguish and some of the samples come from yellow traps where the insects can be in a bad condition, complicating morphological identification. Additionally, even specialists in insect morphology cannot identify some of these species. For seven of those libraries (whiteflies/aphids), the species ID results in an identification to a deeper taxonomic level. Two of them (Bactrocera and Zeugodacus are synonyms and thus we changed all to Bactrocera to eliminate confusion. 1 of those are assumed Bactrocera dorsalis/ invadens. These two species cannot be distinguished by morphology. Species ID with the described method presents an improved resolution compared to the existing methodology. The remaining 2, Delia platura/antiqua- Botanophila fugax can hardly be distinguished by morphology, thus the species ID assigned by the presented Nanopore sequencing methodology presents a method where these species/subspecies can be identified without doubt.

To reflect these thoughts we added the sentence

“Some of the samples (aphids, Tenthredinidae, whiteflies) could be assigned to a deeper taxonomic level with the novel approach while some of the samples (Bactrocera, Delia) resulted in a different species than initially assumed based solely on morphology.”

In Line242 of the manuscript.

And the footnote

^c^ Assumed Sample Species: This is the educated guess of non-taxonomist layman at the airport or entomologists based solely on morphology

As a footnote to Table S1

A final comment about the application of the method to real samples. The manuscript uses artificial single species libraries and the bioinformatic pipeline is intended to provide one species per sample. But what would happen if the original sample contained more than one insect species? Ideally, the method should provide all of them, but would it? In addition, if the sample contained two phylogenetically close species (e.g. two species of Bactrocera) would their reads map in different contigs or would they be mixed? I understand that these questions can be difficult to handle at this stage, but the authors might consider adding their reflections to the discussion. Depending on their willingness, they could simulate two-species libraries by merging the fastq files of two single-species libraries, run the entire pipeline using the new fastq file, and see what happens.

We agree with the reviewer that mixed samples is a completely different story. The whole body of work was explicitly developed for insects where single unknown individuals can be sampled (as single larvae, adults, or body parts such as legs, torsos or wings). We tested expansion to other kingdoms with bacteria and fungi knowing that we would only pick up the major species/pathogen present which for a lot of situations in pathogen detection is sufficient. While Nanopore sequencing has been shown to identify mixtures, the bioinformatics approach would be completely different, similar to a metagenomic sequence classification approach as in Kraken (https://genomebiology.biomedcentral.com/articles/10.1186/gb-2014-15-3-r46) also implemented for example in Oxfords own WIMP.

To reflect these thoughts and not raising false hopes we added the statement

“The presented work has explicitly been developed for the identification of single unknown individuals. While the Nanopore sequencing approach can be applied to research questions where mixtures of species are present a completely different bioinformatics approach would be chosen similar to a metagenomic sequence classification as in Kraken. “

in Line 329 of the manuscript

Reviewer #2: This manuscript is introducing a WGA based protocol utilizing ON MinION sequencing to improve DNA-based species identification. the methods is described very well, sufficient details on all steps are provided. My major point of criticism is that the discussion section lacks several aspects mentioned in the results e.g., on the presence of genes of interest such as insecticide resistance genes, and the presence of arthropod-infecting
viruses and endosymbiotic bacteria.

We have added the statement

“This allows answering questions with relevance beyond simply identifying the species, such as the identification of resistance genes informing efficient selection of plant protection antibiotics or fungicides as in apple fire blight [29, 30] or apple scab prevention [31]. Similarly, the presence of previously described insecticide resistance genes can inform the efficient application of insecticides [32, 33]. Additionally, proposed arbovirus surveillance schemes using deep sequencing [34] could profit from the “sequence anywhere” capability of Oxford Nanopores sequencing platform.”

To line 317 of the discussion

for a protocol description such as this it would be also very interesting to discuss the reference libraries used – where they sufficient?

We feel that the manuscript shows the sufficiency of the reference libraries for the sought task of species identification.

If not, how to improve that, where are the gaps, etc. ; or what about the pipelines you used?

To answer this comment and in order to answer a point from reviewer 1 we have added a statement describing how to handle mixed samples for more complex analyses in line 329

and we had already described in the first draft in line 339 that for answering questions in invasion biology, much larger reference databases would need to be constructed.

Where they useful, what could be alternatives also given that Geneious is not cheap?

The described approach was useful for the given task. Geneious comes with a price, true, but most of their applied algorithms are published and freely available bioinformatics tools. So anybody with some coding skills can reproduce the analyses without licensing fees. If too tedious an approach using Kraken as described in line 330 would certainly also work for single unknown individuals.

Minor things:
L29 - 658 basepairs if you refer to COI- done, now L34
L58 - is needed not are needed- done, now é64
L64 - shown not proofed- done
L125 - add a 'the' between extractions and DNA- done, now L131
L128 - add the missing )- done, L134
L214 – How did you compensate for retained sequences of prior runs? MinION flow cells are known to retain up to 15% of former runs even if their wash kits are used. Did you see a difference (or overlap) between runs of reused flowcells? This should also be briefly discussed.

This has been true in the past. With the addition of a so called “nuclease flush” to their standard wash protocol and later on including the nucleases into their normal wash chemistry with WSH003 (around 2018) and WSH004 (early 2020) old libraries are degraded on the chip. ONT has shown that the old library contributes less than 0.05% to the total amount of reads. (See link below)

<https://community.nanoporetech.com/posts/flow-cell-wash-kit-exp-ws>

We wrongly described having used the WSH002 kit. In fact, we have used the WSH003 kit, including the nucleases, throughout the study and have changed this in the manuscript in line 149

L239 – table caption talks about Nematode data, but none is shown.

Thanks for finding this error. This line must have slipped somehow during manuscript preparation. The table is now updated.

L309 – remove “should essentially enable to” and use ‘could’- done, now L329
L314 – Interest not interests- done, now L336
